# Deploying Experienced Utility in Health Economic Evaluation: A Quantitative Study

**DOI:** 10.3390/jmahp13030043

**Published:** 2025-08-28

**Authors:** Damien S. E. Broekharst, Sjaak Bloem, Robert J. Blomme, Edward A. G. Groenland, Patrick P. T. Jeurissen, Michel van Agthoven

**Affiliations:** 1 Center for Marketing & Supply Chain Management, Nyenrode Business University, 3621 BG Breukelen, The Netherlands; 2 Center for Strategy, Organization & Leadership, Nyenrode Business University, 3621 BG Breukelen, The Netherlands; 3 IQ Healthcare, Radboud University Medical Centre, 6525 GA Nijmegen, The Netherlands; 4 Janssen-Cilag B.V., Johnson & Johnson, 4837 DS Breda, The Netherlands

**Keywords:** expected utility, experienced utility, health utility, health preference, health experience

## Abstract

Background: Expected utility has been deployed in order to predict health behaviour in health economic evaluation. However, only limited variance in health behaviour is explained by this construct. This limited explained variance is often attributed to the dubious foundational postulates underlying the construct (e.g., absolute rationality, complete information, fixed preferences). Due to these limitations it has been hypothesized that substituting or complementing expected utility with experienced utility may enhance predictions of health behaviour. As this hypothesis has not yet been subjected to empirical scrutiny, this study examines if deployment of experienced utility or expected utility and experienced utility combined enhances predictions of health behaviour relative to expected utility separately. Methods: Online questionnaires were distributed across a panel of Dutch citizens (N = 2550). The questionnaire includes items and scales on sample characteristics, expected utility, experienced utility and health behaviour. Data analysis was conducted by employing descriptive, reliability, validity and model statistics. Results: Experienced utility has a significant direct effect on health behaviour that is stronger than expected utility. Experienced utility also explains more variance in health behaviour than expected utility. Expected utility and experienced utility combined have a significant direct and indirect effect on health behaviour that is stronger than each type of utility separately. Expected utility and experienced utility combined also explain more variance in health behaviour than each type of utility separately. Conclusions: Deploying experienced utility separately or in combination with expected utility in health economic evaluation seems pertinent as it has considerable impact on health behaviour and may provide health economists with an even sturdier foundation for conducting health economic evaluation.

## 1. Introduction

In recent decades, expected utility has secured a firm foothold within the discipline of health economics serving as a central pillar of health economic evaluation [1]. Expected utility in health economic evaluation denotes the weighted preference for a specific health outcome expressed on a cardinal scale where 1.0 demonstrates full health, 0.0 is commensurate with death and values below 0.0 delineate health states worse than death [1]. Expected utility in health economic evaluation is currently measured using generic or specific health status or quality of life questionnaires (e.g., SF-6D, EQ-5D-3L, EQ-5D-5L) [2,3]. The outcomes of these questionnaires are subsequently valued by classical economic valuation methods (e.g., time-trade-off method, standard gamble approach, visual analogue scale) of which the time-trade-off method is most often deployed [4,5,6]. This preference-based valuation approach elicits expected utility values by determining the proportion of remaining life expectancy an individual is willing to forgo in order to avert persisting in a sub perfect health state [4,5,6]. The expected utility values produced by this determinative exercise are assumed to be accurate predictors of actual health behaviour, which denotes the health-related practices that impact the overall health of an individual [5,6,7,8].

However, an ever expanding cohort of behavioural economists and psychologists find fault with this assumption as they argue that its theoretical axioms are not supported by scientific literature and can only be accepted under particular circumstances making expected utility an imprecise predictor of health behaviour [8,9]. Several studies indicate that the aforementioned assumption can only be accepted if expected utility values are based on absolute rationality, while research shows that they are often based on bounded rationality [9,10]. Scientific literature also suggests that this assumption can only be accepted if expected utility values are based on complete information, while research reveals that they are often based on incomplete information [9]. Research further shows that this assumption can only be accepted if expected utility values are stable over time, while research suggests that they are flexible and dynamic over time [9]. In order to account for these apparent shortcomings it has been hypothesized by the apparent critics and detractors of expected utility, such as Daniel Kahneman and his contemporaries, that substituting or complementing expected utility in health economic evaluation with actual experienced utility may enhance predictions of health behaviour [9,10,11].

Experienced utility emerged from psychological literature and refers to the subjective value, expressed in terms of perceived pain or pleasure, that is attributed to the cognitive, affective and conative experience of one’s own physical, mental and social health state, which is determined through self-reflection and interaction with significant others [12,13]. This conceptualization of utility and its subsequent measurement allows for the ex-post elicitation of individual perceptions regarding realized health outcomes, which can also be deployed to predict future health behaviour [14]. However, to our knowledge, no attempts have been levied at measuring and valuing experienced utility in health economic evaluation or deploying it to substitute or complement expected utility for the purpose of predicting health behaviour as hypothesized by Daniel Kahneman and his contemporaries [9,10,11]. Therefore, this study examines if the deployment of experienced utility or a combination of expected utility and experienced utility in health economic evaluation enhances the prediction of health behaviour relative to the traditional deployment of expected utility. The findings of this study hold potential methodological significance as the influence of experienced utility on health behaviour could justify its integration into Markov models to derive conditional transition probabilities and generate dynamic quality-adjusted life years (QALYs). Furthermore, the results may have procedural relevance informing the design, evaluation and appraisal of health behaviour interventions and public policy initiatives as well as guiding decisions on resource allocation.

## 2. Methods

### 2.1. Design, Procedure and Participants

In order to address the research question outlined above, a quantitative research design employing questionnaires was implemented. The questionnaires were administered online to a large panel of Dutch citizens maintained by the IPSOS research agency. Panel members were invited via email and provided with the necessary information along with a formal request for consent. Only individuals aged 18 years or older who consented to the use of their responses in future research were eligible to participate. The administered questionnaires included several items capturing sample characteristics followed by multiple measurement instruments assessing expected utility, experienced utility and health behaviour. Data collection for this study occurred between September and December 2021.

### 2.2. Questionnaire, Items and Scales

Sample characteristics were described using multiple singular items on age, gender, health status, living area, residential region, educational level and annual income. These items were measured on nominal scales with dichotomous response categories or ordinal scales using ascending response categories. Expected utility was determined using the EuroQol Five-dimensions Five-level (EQ-5D-5L) instrument in combination with the preference-based time-trade-off method [15,16]. This instrument and validation method are an important standard for the elicitation of expected utility values in health economic evaluation [15,16]. The EQ-5D-5L instrument consists of five dimensions, namely mobility, self-care, usual activities, pain and discomfort and anxiety and depression (Supplementary Materials) [15,16]. These dimensions were measured with a 5-point scale creating generic health state profiles that were subsequently linked to standard collective expected utility values derived from a nationwide Dutch time-trade-off study [17]. In such studies, expected utility values of health states are determined by inquiring among the public how much lifespan one would trade-off to avoid a suboptimal health state [17]. Experienced utility was measured using Subjective Health Experience (SHE) ladders [18,19,20,21]. The SHE ladder is one of the few validated instruments that is able to capture the experienced utility of health states as perceived by individuals [18,19,20,21]. The SHE ladders were administered in order to measure experienced utility with regard to physical health, psychological health, social health and general health pertaining to the previous month (Supplementary Materials) [18,19,20,21]. Scores were determined by selecting one of the 11 rungs on the SHE ladder with 0 representing the worst day and 11 representing the best day experienced in the previous month [18,19,20,21]. Such ladder and other visual analogue scales can be deployed to directly generate experienced utility values [22,23,24,25]. Health behaviour was measured using an instrument based on the BRAVO dimensions [26]. These BRAVO dimensions are often deployed and show validity across different populations and countries [26]. This instrument consists of six dimensions, videlicet, exercise, nutrition, rest, smoking, alcohol use and general health (Supplementary Materials) [26]. These dimensions were measured on a 6-point Likert scale with participants rating their agreement from 1 = fully disagree to 6 = fully agree [26].

### 2.3. Statistical Analyses

Sample characteristics were summarized using descriptive statistics. Continuous variables were reported as means, while categorical variables were expressed as percentages. Questionnaire characteristics were evaluated using indices of construct reliability, construct validity, convergent validity and discriminant validity. Construct reliability of the instruments was assessed via Cronbach’s alpha (α), rho_a and rho_c coefficients with values exceeding the 0.70 threshold indicative of acceptable reliability [27]. The construct validity of the instruments was determined by performing a factor analysis in which the factorial structure of these scales was confirmed and optimized by removing double or triple loading items [27]. The convergent validity of the instruments was examined using the average variance extracted (AVE) coefficient, which can be deemed sufficient if its value exceeds the 0.50 threshold [27]. The discriminant validity of the instruments was analysed and described using the heterotrait-monotrait (HTMT) ratio that was deemed adequate if its value was below the 0.90 threshold [27]. Model characteristics were analysed through partial least squares structural equation modelling (PLS-SEM) as this modeling approach requires few assumptions about data distribution and reports results in terms of effect sizes, significance levels, and explained variance [27]. The effect sizes of relationships between model variables are interpreted using standardized Beta-coefficients (β), which can be deemed small below 0.30, medium between 0.30 and 0.50 and large above the 0.50 threshold [27]. The significance levels of the aforementioned relationships between model variables are analysed using *p*-values, which are deemed significant if they are lower than the 0.05 threshold [27]. The explained variance of independent variables in the dependent variable was described using R-squared (R^2^) coefficients, which can be deemed weak below 0.30, moderate between 0.30 and 0.50 and substantial above the 0.50 threshold [27]. For the purposes of clarity and comprehensibility only the general models have been presented as segmentation using available control variables (e.g., age, gender, health status) provided limited additional insight except for some small deviations. Sample characteristics were extracted using software package IBM SPSS Statistics Version 27 [28]. Questionnaire and model characteristics were analysed using software package SmartPLS Version 4.0 [27].

## 3. Results

### 3.1. Sample Characteristics

A total of 2550 panel members constituted the final sample. The final sample closely resembled the general population of the Netherlands in terms of age, gender, health status, living area, residential region, educational level and annual income, while also showing a slight overrepresentation of individuals from more urbanized areas [29,30,31,32,33,34,35,36]. An overview of the sample characteristics is presented in Table 1.

### 3.2. Questionnaire Characteristics

The instruments assessing experienced utility (α = 0.86; rho_a = 0.86; rho_c = 0.90) and health behaviour (α = 0.72; rho_a = 0.79; rho_c = 0.82) can be deemed reliable as each reported reliability coefficient surpassed the standard 0.70 threshold. The instrument assessing experienced utility suggested sufficient construct validity as all items loaded on a single component, while the instrument assessing health behaviour further suggested sufficient construct validity after excluding two items (i.e., ‘smoking’, ‘alcohol use’). The instruments assessing experienced utility (AVE = 0.70) and health behaviour (AVE = 0.54) suggested sufficient convergent validity as the average variance extracted surpassed the standard 0.50 threshold. The instruments assessing experienced utility and expected utility (HTMT = 0.60), the instruments assessing expected utility and health behaviour (HTMT = 0.53), and the instruments assessing experienced utility and health behaviour (HTMT = 0.68) all suggested sufficient discriminant validity as the heterotrait-monotrait ratios persisted below the standard 0.90 threshold. For the instrument measuring expected utility, reliability and validity could not be evaluated as it comprises individual utility values and lacks multidimensionality. Nevertheless, based on the metrics reported above the measurement instruments employed in this study could be regarded as both reliable and valid.

### 3.3. Model Characteristics

Three statistical models have been established in order to explore whether the deployment of experienced utility or a combination of expected utility and experienced utility in health economic evaluation enhances the prediction of health behaviour as compared to the traditional deployment of expected utility.

### 3.4. Expected Utility and Health Behaviour

**Figure 1:**
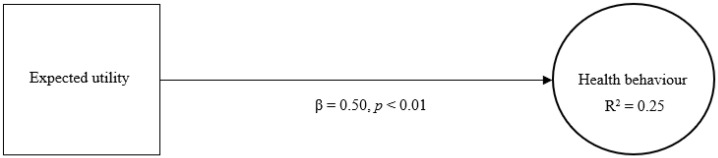
Expected utility and health behaviour. Legend: β = standardized beta coefficient; *p* = significance level; R^2^ = explained variance.

The first model determines the effect and explained variance of expected utility with regard to health behaviour (Figure 1). According to this model, expected utility (β = 0.50, *p* < 0.01) demonstrates a significant and relatively strong direct effect on health behaviour, providing it with acceptable predictive capacity. Moreover, the model shows that experienced utility accounts for a limited degree of variance in health behaviour (R^2^ = 0.25).

### 3.5. Experienced Utility and Health Behaviour

**Figure 2:**
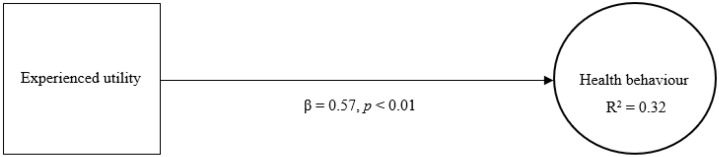
Experienced utility and health behaviour. Legend: β = standardized beta coefficient; *p* = significance level; R^2^ = explained variance.

The second model determines the effect and explained variance of experienced utility with regard to health behaviour (Figure 2). According to this model, experienced utility (β = 0.57, *p* < 0.01) demonstrates a significant and even more pronounced direct effect on health behaviour relative to expected utility providing it with meaningful predictive capacity. Moreover, the model shows that experienced utility accounts for a higher degree of variance in health behaviour (R^2^ = 0.32) relative to expected utility.

### 3.6. Expected Utility, Experienced Utility and Health Behaviour

**Figure 3:**
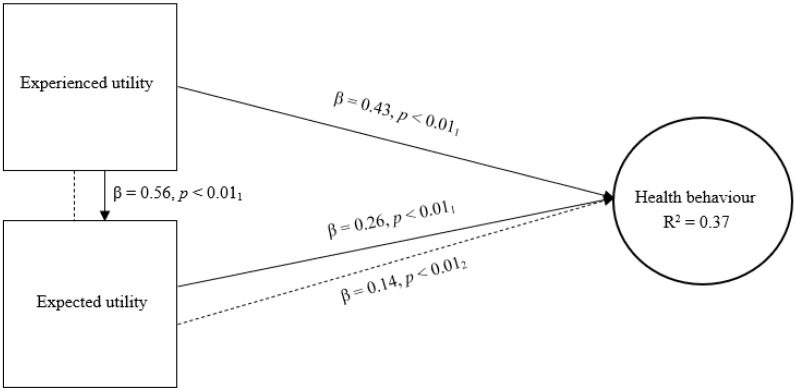
Expected utility, experienced utility and health behaviour. Legend: β = standardized beta coefficient; *p* = significance level; R^2^ = explained variance; _1_ = direct effect; _2_ = indirect effect.

The third model determines the combined effect and explained variance of expected utility and experienced utility with regard to health behaviour (Figure 3). This model indicates that the combined direct effect of expected utility (β = 0.26, *p* < 0.01) and experienced utility (β = 0.43, *p* < 0.01) is significant and stronger than each type of utility separately providing this combination with considerable predictive power. Additionally, this model suggests a small significant indirect effect of experienced utility on health behaviour through expected utility (β = 0.14, *p* < 0.01), which demonstrates a partial mediation effect. This model further shows that expected utility and experienced utility combined explain more variance in health behaviour (R^2^ = 0.37) than each separately.

## 4. Discussion

This study examines if the deployment of experienced utility or a combination of expected utility and experienced utility in health economic evaluation enhances the prediction of health behaviour relative to the traditional deployment of expected utility. The findings show that experienced utility has a significant direct effect on health behaviour that is even stronger than that of expected utility. The findings also suggest that experienced utility explains more variance in health behaviour than expected utility. The results further show that expected utility and experienced utility combined have a significant direct and indirect effect on health behaviour that is stronger than each type of utility separately. The results finally suggest that expected utility and experienced utility combined explain more variance in health behaviour than each type of utility separately.

In previous research expected utility has been assumed to be an accurate predictor of health behaviour [5,6,7,8]. This study confirms the aforementioned assumption as expected utility has a significant and rather strong effect on and explains certain variance in health behaviour. However, this study also shows that the explained variance of expected utility in health behaviour is not particularly high. This rather limited explained variance might be caused by the bounded rationality, limited information and inflexible preferences underlying the largely hypothetical and somewhat imprecise preference-based valuation method on which expected utility is predicated [8,9]. In response to the perceived shortcomings of expected utility multiple authors have postulated that deploying experienced utility might further enhance predictions of health behaviour [9,10,11]. This study confirms the aforementioned hypothesis as experienced utility has a larger effect on and explains more variance in health behaviour than expected utility. This greater effect size and explained variance may result from the direct measurement of the real-life experiences underpinning experienced utility, rather than relying on hypothetical and imprecise proxies as is typical in the case of expected utility [12,14]. Notwithstanding, certain authors also argue that expected utility and experienced utility have their own distinct impact on health behaviour proposing that their conjunctive use might enhance predictions of health behaviour [12,14]. This study confirms this proposition as the combined effect of expected utility and experienced utility on health behaviour is larger than that of each type of utility separately as is the case for explained variance. This increased effect size and explained variance might be due to the conjunctive use of different utility instruments that complement, reinforce and embellish as well as counterbalance, rectify and correct each other.

Nevertheless, it should be noted that the conjunctive use of expected utility and experienced utility could provide certain practical problems as it could be costly and time-consuming to measure both types of utility in the domain of health economic evaluation. However, in particular situations it could be especially worthwhile to deploy both types of utility, such as in the evaluation of treatments for chronic diseases that strongly interfere with the daily experiences of patients (e.g., Parkinson’s disease, irritable bowel syndrome, rheumatoid arthritis). In addition, it should be remarked that the combined explained variance of expected utility and experienced utility in health behaviour could still only be considered moderate. This suggests that existing concepts might need to be operationalized differently or that other concepts may need to be included. One might suggest that expected utility could be operationalized in terms of an individual’s realistic approximation of their future health state instead of an individual’s hypothetical preference for their future health state [37,38]. One might also suggest that the concept of procedural utility could be added to the utilitarian dichotomy presented in this study as both expected utility and experienced utility emphasize future or past health states without considering the process of obtaining such health states [39,40].

## 5. Strengths and Limitations

This study has several strengths as well as limitations. One limitation of this study lies in the observable skew of the final sample towards urbanized respondents evoking the potential misrepresentation and distortion of findings. Another limitation of this study arises from its exclusively Dutch cohort and context potentially jeopardizing the generalizability and interpretability of findings. An important strength of this study relates to the considerable sample size that has been obtained improving the representativity and generalizability of results. An additional strength of this study emerges from the robust and dependable measurement instruments enhancing the accuracy and integrity of results.

## 6. Practical Implications

The findings of this study carry several significant implications for practice. The results initially imply that health economic evaluation should not merely rely on conventional measurement and valuation studies resulting from standard economic methods and approaches. This observation subsequently implies that alternative methods and approaches (e.g., evidence-informed deliberative processes, integrated health technology assessment, experienced utility measurement) applied in order to gather real-world evidence should be further explored as this could improve accuracy of and trust in health economic evaluation. Moreover, the findings particularly imply that this improvement may be achieved by applying psychological instruments on experienced utility in health economic evaluations. This can be achieved by incorporating experienced utility into health economic models (e.g., Markov models, microsimulations) using conditional instead of fixed transition probabilities that reflect lived experiences enabling behaviourally informed and dynamically responsive modelling that generates QALY estimates evolving with changes in quality of life and behaviour. Furthermore, the integration of experienced utility into health policy development may have procedural relevance informing the design, evaluation and appraisal of health behaviour interventions and public policy initiatives as well as guiding decisions on resource allocation.

## 7. Future Research

There are four key directions for further investigation that might be pursued. The first direction for further investigation pertains to exploring different conceptualizations and operationalizations of the concepts deployed in this study in order to improve measurement accuracy (i.e., expected utility, experienced utility, health behaviour). The second direction for further investigation pertains to investigating other possible concepts that might enhance the prediction of health behaviour in addition to expected utility and experienced utility (e.g., procedural utility). The third direction for further investigation pertains to repeating this research in other countries and populations in order to improve generalizability and interpretability of findings. The fourth direction for further investigation pertains to examining the transactional costs of measuring experienced utility separately or expected utility and experienced utility combined.

## 8. Conclusions

Given the findings of this study, it can be concluded that expected utility does have an effect on and explains a certain amount of variance in health behaviour. However, the impact of expected utility is not particularly decisive encouraging the introduction of other concepts that might contribute to the accuracy of the prediction of health behaviour. Experienced utility seems to be a pertinent substitute of or addition to the already deployed expected utility as this concept explains a considerable part of the variance in health behaviour. Therefore, deploying experienced utility either separately or in combination with expected utility may provide health economists with an even sturdier foundation for conducting health economic evaluation albeit after deliberate reflection on the pragmatic consequences and accompanying challenges. Nevertheless, other concepts that may contribute to the accuracy of predictions regarding health behaviour should also be considered and examined.

## Figures and Tables

**Table 1:** Sample description.

Variables	
Age	$\bar{x}$ 49.3 years (18–89 years)
Gender	
Male	48.6%
Female	51.4%
Health status	
Healthy	37.2%
One disease	27.9%
Comorbidities	34.9%
Living area	
City	56.8%
Suburb	34.3%
Rural	8.9%
Residential region	
North Netherlands	10.1%
East Netherlands	21.1%
West Netherlands	48.1%
South Netherlands	20.7%
Education level	
Low	31.5%
Average	29.3%
Higher	38.9%
Unknown	0.4%
Annual income	
<€36,500	35.2%
€36,500–€73,000	35.8%
>€73,000	14.6%
Unknown	14.4%

## Data Availability

The original contributions presented in this study are included in the article. Further inquiries can be directed to the corresponding author.

## Supplementary Materials

The following supporting information can be downloaded at: https://www.mdpi.com/article/10.3390/jmahp13030043/s1, Table S1: EQ-5D-5L questionnaire; Table S2: SHE questionnaire; Table S3: BRAVO questionnaire.
