# Supplementary material for: Deploying Experienced Utility in Health Economic Evaluation: A Quantitative Study
Source: J Mark Access Health Policy. 2025 Aug 28;13(3):43. doi: 10.3390/jmahp13030043 (PMC12452304; doi:10.3390/jmahp13030043)
Supplement: Supplementary file 1 [file jmahp-13-00043-s001.zip › jmahp-3771272-supplementary.pdf]

Table S1. EQ-5D-5L questionnaire

Under each heading, please tick the ONE box that best describes your health TODAY.

MOBILITY

I have no problems in walking about	❑
I have slight problems in walking about	❑
I have moderate problems in walking about	❑
I have severe problems in walking about	❑
I am unable to walk about	❑

SELF-CARE

I have no problems washing or dressing myself	❑
I have slight problems washing or dressing myself	❑
I have moderate problems washing or dressing myself	❑
I have severe problems washing or dressing myself	❑
I am unable to wash or dress myself	❑

USUAL ACTIVITIES *(e.g. work, study, housework, family or leisure activities)*

I have no problems doing my usual activities	❑
I have slight problems doing my usual activities	❑
I have moderate problems doing my usual activities	❑
I have severe problems doing my usual activities	❑
I am unable to do my usual activities	❑

PAIN / DISCOMFORT

I have no pain or discomfort	❑
I have slight pain or discomfort	❑
I have moderate pain or discomfort	❑
I have severe pain or discomfort	❑
I have extreme pain or discomfort	❑

ANXIETY / DEPRESSION

I am not anxious or depressed	❑
I am slightly anxious or depressed	❑
I am moderately anxious or depressed	❑
I am severely anxious or depressed	❑
I am extremely anxious or depressed	❑

Sample

Table S2. SHE questionnaire

**What is your experience regarding your physical health in the past period?**	Your experience (on average) over the **last 4 weeks**:
10 – Recall the **best** period	
9	
8	
7	
6	
5	
4	
3	
2	
1	
0 - Recall the **worst** period	

**What is your experience regarding your mental health in the past period?**	Your experience (on average) over the **last 4 weeks**:
10 – Recall the **best** period	
9	
8	
7	
6	
5	
4	
3	
2	
1	
0 - Recall the **worst** period	

**What is your experience regarding your social life in the past period?**	Your experience (on average) over the **last 4 weeks**:
10 – Recall the **best** period	
9	
8	
7	
6	
5	
4	
3	
2	
1	
0 – Recall the **worst** period	

**What is your experience regarding your general health (physical, mental, social) in the past period?**	Your experience (on average) over the **last 4 weeks**:
10 – Reacall the **best** period	
9	
8	
7	
6	
5	
4	
3	
2	
1	
0 - Recall the **worst** period	

Table S3. BRAVO questionnaire

	1 – Fully disagree	2	3	4	5	6 – Fully agree
I engage in frequent physical activity						
I smoke regularly						
I consume alcohol regularly						
I follow a healthy and balanced diet						
I ensure I get sufficient rest						
I consistently engage in behaviours that support good overall health						
